# Prevalence of overweight and obesity among saudi children: A comparison of two widely used international standards and the national growth references

**DOI:** 10.3389/fendo.2022.954755

**Published:** 2022-08-08

**Authors:** Hazzaa M. Al-Hazzaa, Amani A. Alrasheedi, Rayan A. Alsulaimani, Laura Jabri, Abdulrahman M. Alhowikan, Maha H. Alhussain, Rowaedh A. Bawaked, Saleh A. Alqahtani

**Affiliations:** ^1^ Lifestyle and Health Research Center, Health Sciences Research Center, Princess Nourah Bint Abdulrahman University, Riyadh, Saudi Arabia; ^2^ Department of Food and Nutrition, Faculty of Human Sciences and Design, King Abdulaziz University, Jeddah, Saudi Arabia; ^3^ Department of Pharmacology, Faculty of Medicine, King Abdulaziz University, Jeddah, Saudi Arabia; ^4^ American International School of Jeddah, Jeddah, Saudi Arabia; ^5^ Department of Physiology, College of Medicine, King Saud University, Riyadh, Saudi Arabia; ^6^ Department of Food Science & Nutrition, College of Foods & Agricultural Sciences, King Saud University, Riyadh, Saudi Arabia; ^7^ Department of Public Health, College of Health Sciences, Saudi Electronic University, Riyadh, Saudi Arabia; ^8^ Division of Gastroenterology and Hepatology, Johns Hopkins University, Baltimore, MD, United States; ^9^ King Faisal Specialist Hospital & Research Center, Riyadh, Saudi Arabia

**Keywords:** body mass index (BMI), children, International Obesity Task Force (IOTF), overweight, obesity, sociodemographic, underweight, World Health Organization (WHO)

## Abstract

**Objective:**

To compare three body mass index (BMI) classifications that are used to assess the prevalence of overweight and obesity among Saudi children aged 6–13 years: the International Obesity Task Force (IOTF) age and gender cutoffs, the World Health Organization (WHO) growth references for school-aged children, and the Saudi (KSA) national growth references.

**Methods:**

The sample comprised 2,169 children (52.5% girls) derived from two cross-sectional studies conducted in Riyadh and Jeddah during the 2017 and 2019 school years, respectively. Body weight and height were measured, and BMI was calculated.

**Results:**

The proportions (%) of the participants who were classified as underweight, overweight, and obese varied according to the reference used: IOTF reference (13.8, 18.4, and 12.7), WHO reference (17.2, 19.1, and 18.9), and KSA reference (7.0, 22.4, and 9.3), respectively, indicating higher values for overweight and obesity prevalence when the WHO references were used. Kappa agreement measures between the three references were found to be high, with the coefficients ranging from 0.936 (between the IOTF and KSA references) to 0.849 (between the IOTF and WHO references). In all three classifications, girls exhibited lower overweight or obesity prevalence than boys. Family income, but not paternal or maternal education, was significantly (*p* = 0.015) associated with overweight/obesity when using the IOTF standards. In addition, having a small family in the house was significantly (*p* < 0.05) associated with obesity, irrespective of the classification system.

**Conclusion:**

Inconsistency was observed when estimating the prevalence of underweight, overweight, and obesity among Saudi children. However, when defining the overall prevalence of overweight plus obesity among Saudi children, the IOTF classification system performed in a similar way to the KSA references (31.1% versus 31.7%) compared to the WHO references (38.0%).

## Background

Childhood obesity is arguably the most serious recent public health challenge (1). Indeed, it is a worldwide public health concern with many major negative consequences (2, 3). Being overweight or obese in childhood and adolescence is associated with greater risk and earlier onset of chronic disorders, such as type 2 diabetes (2). Childhood and adolescent obesity have been shown to have adverse psychosocial consequences and lower educational attainment (3), and excess body weight in childhood and adolescence is more likely to lead to lifelong overweight and obesity (4, 5). Additionally, in a recent eight-country study, the economic impacts of obesity were found to be substantial in all eight countries, regardless of economic or geographical setting, ranging from 0.8% of gross domestic product (GDP) in India to 2.4% in Saudi Arabia (6).

The age-standardized prevalence of obesity increased globally from 0.7% (0.4–1.2%) in 1975 to 5.6% (4.8–6.5%) in 2016 in girls, and from 0.9% (0.5–1.3%) in 1975 to 7.8% (6.7–9.1%) in 2016 in boys (7). However, the prevalence of obesity was about 20% or more in several countries and regions, such as Polynesia and Micronesia, the Middle East and North Africa, the Caribbean, and the USA (7). In Saudi Arabia, the percentage of children classified as overweight or obese has significantly increased in the past two decades (8–11). A recent review of overweight and obesity among Saudi children found that the ranges of overweight and obesity were larger in boys (19.3–35.6%) than in girls (11.8–19.2%) (10).

The implementation of school-based BMI measurement has become popular as a potential approach to addressing overweight and obesity among youth (12). However, defining overweight and obesity in children and adolescents is not as straightforward as it is in adults. Usually, the International Obesity Task Force (IOTF) BMI cutoff values are used, which are set using data collected from six countries: Singapore, the Netherlands, Brazil, Hong Kong, the UK, and the USA (13). Another approach is to use the World Health Organization’s (WHO) reference standards for children and adolescents aged 5–19 years, which are based on weight-for-height Z-scores (14). Both of these methods for defining overweight and obesity in children are generally valid; however, they often produce different results. Within the same population, the IOTF reference tends to yield the lowest values, and the WHO reference tends to yield the highest values (15–20). For instance, a study involving Saudi national data reported major differences between the use of Saudi growth charts of weight for age (21) and the WHO reference (14). The study concluded that the utilization of the WHO standards in Saudi Arabia, and possibly similar countries, increases the reported prevalence of undernutrition, stunting, and wasting, which potentially leads to unnecessary referrals, investigations, and parental concern (21).

Thus, it is challenging to determine the actual prevalence of underweight, overweight, and obesity among children and adolescents when such inconsistency exists among the most common international classification systems (13, 14). Therefore, the present study aimed to compare the three classifications that are used to assess overweight and obesity among Saudi children aged 6–13 years, namely the IOTF age and gender cutoff values (13), WHO growth references for school-aged children (14), and the Saudi (KSA) national growth references (22).

## Methods

### Population and sample

The population in this study consisted of healthy students of both sexes aged 6–13 years who attended public and private primary schools in two major cities in Saudi Arabia. The sample was drawn from two cross-sectional studies conducted in Riyadh and Jeddah during the 2017 and 2019 school years, respectively (23, 24). Riyadh and Jeddah are the first- and second-most populated cities in Saudi Arabia, respectively. The two cities are also composed of a multiethnic population coming from all parts of the country. All healthy Saudi children enrolled in primary schools from grades 1–6 during the study periods were eligible for inclusion in the study. Detailed descriptions of the study design and sample selection were previously published (23, 24).

Briefly, the sample size was calculated assuming that the population proportion would yield the maximum possible sample size required (proportion = 0.50), with a 95% confidence level and a 4% margin of error. An additional 20% of participants were added to account for non-responders or missing data. A representative random sample was chosen from schools in each selected city using a multistage stratified cluster sampling technique. Stratification was based on sex (boys’ and girls’ schools are segregated in Saudi Arabia), major geographical location (east, west, north, and south), and type of school (public versus private). Participating children were selected from primary schools relative to the actual number of students in public and private schools in each city. Within each area, one private and two public schools were randomly selected. Then, classes were randomly selected from each of the six grades. All Saudi students in the designated classes were invited to participate in the study.

### Anthropometric measurement and BMI classification

Measurements of body weight (to the nearest 100 g) and standing height (to the nearest 0.1 cm) were performed at the schools by trained researchers using calibrated portable scales (Seca 869, UK) and height measuring rods, respectively. Students wore minimal clothing and no shoes when the measurements were taken. Body mass index (BMI) was computed as the ratio of weight in kilograms divided by the squared height in meters.

The outcome measure in the present study was the classification of the BMI data into the categories of underweight, normal weight, overweight, and obesity. Two commonly used international reference standards (cutoff values) were used to classify the BMI data. The first was the extended IOTF age- and sex-specific BMI cutoff reference standards, which are based on data from children and adolescents in six countries: Brazil, Hong Kong, the Netherlands, Singapore, the UK, and the USA (13). The second was the WHO growth references for school-aged children and adolescents (5–19 years), published in 2007 and based on weight-for-height Z-scores (14). For comparison, we also included the KSA national growth references (from underweight to obesity), which were calculated from the z−scores of BMI for age for children and adolescents aged 5–18 years (22). The IOTF references provide percentile cut-offs corresponding to a BMI of 18.5, 25, and 30 kg/m2 at 18 years of age for underweight, over weight, and obesity, respectively (13). The prevalence of underweight, overweight, and obese are defined by the WHO (14) and the KSA (22) cut-off values as BMI-for-age less than 2 standard deviation (SD) scores below the mean, greater than 1 SD above the mean, and greater than 2 SDs above the mean, respectively. All three classification systems used are all based on the lambda (L), mu (M), and sigma (S) method (25, 26). The LMS parameters correspond to median BMI (M), coefficient of variation (S), and the power in the Box–Cox transformation (L), which transforms the data so that it closely resembles a normal distribution (25, 26).

### Ethical approval

Ethical approval was obtained from the Institutional Review Board (IRB) at King Saud University, Riyadh (IRB Log Number: 17/0064/IRB) and Princess Nourah bint Abdulrahman University, Riyadh (IRB Log Number: 19-0014). The research procedures were conducted in accordance with the principles stated in the Declaration of Helsinki. Written informed consent was obtained from all parents/guardians of the participating children. In addition, approval for conducting this research in schools was attained from the Riyadh and Jeddah directorates of schools, the Ministry of Education, and the principals of the selected schools.

### Statistical analysis

Data were entered into an SPSS data file, checked for accuracy, cleaned, and analyzed using the IBM-SPSS software, version 22 (Chicago, IL, USA). Descriptive statistics were obtained for the selected variables and reported as means and standard deviations or percentages for continuous or categorical variables, respectively. Differences between boys and girls in selected measurements were tested using the t-test for independent samples. Chi-square tests of proportions were used to test the differences in BMI classifications (prevalence rates) based on the IOTF, WHO, or KSA national growth references relative to selected sociodemographic factors. Kappa agreement measures for the whole sample were assessed between the three reference standards. Logistic regression analysis, adjusted for age, was used to test the associations of selected sociodemographic variables with overweight/obesity versus non-overweight/non-obesity among Saudi children. Furthermore, Spearman’s rho correlation coefficients, while controlling for age, were calculated between obesity indices, based on the IOTF, WHO, and KSA reference standards, and selected variables. The alpha level was set at 0.05, and a *p*-value less than the alpha level was considered significant.

## Results

**Table 1** presents the descriptive characteristics of the participants. The study included 2,169 participants (52.5% girls) who were between 6 and 13 years of age, and the mean age (SD) was 9.3 (1.7) years. There were significant differences in body weight (*p* = 0.001), BMI (*p* < 0.001), and maternal education (*p* = 0.005) relative to the sex of the participants. However, no significant differences were observed in age, height, paternal education, or family income relative to the sex of the participants.

**Table 1:** Descriptive characteristics of the participants relative to sex.

Variable	All	Boys	Girls	*p*-value *
	N = 2169	N = 1029	N = 1140	
**Age**	9.3 ± 1.7	9.3 ± 1.7	9.3 ± 1.7	0.824
**Body weight** (kg)	34.3 ± 15.4	35.5 ± 18.4	33.2 ± 12.2	0.001
**Body height** (cm)	133.3 ± 11.7	133.5 ± 11.1	133.1 ± 12.2	0.425
**Body mass index** (kg/m**^2^**)	18.7 ± 5.9	19.3 ± 7.5	18.2 ± 4.1	< 0.001
**Father’s education (%)**				0.403
Intermediate or less (¾ 9 years)	12.4	13.5	11.4	
High school	30.2	30.3	30.2	
University degree	46.5	45.0	47.8	
Post graduate degree	10.9	11.2	10.6	
**Mother’s education (%)**				0.005
Intermediate or less (¾ 9 years)	14.3	16.8	12.0	
High school	30.6	29.0	32.1	
University degree	51.3	49.9	52.5	
Post graduate degree	3.8	4.3	3.4	
Family income (%) **				0.239
¾ 10,000 SR	30.1	28.7	31.3	
10,001-20,000 SR	44.9	46.6	43.3	
20,001-30,000 SR	18.6	18.9	18.3	
> 30,001 SR	6.4	5.8	7.1	

Data are means ± standard deviations or percentage.

^*^ T-test for independent samples or Chi Squares tests for the proportion for the differences between boys and girls in continuous or categorical variables, respectively.

**US $ = 3.75 Saudi Riyal (SR).

**Figure 1:**
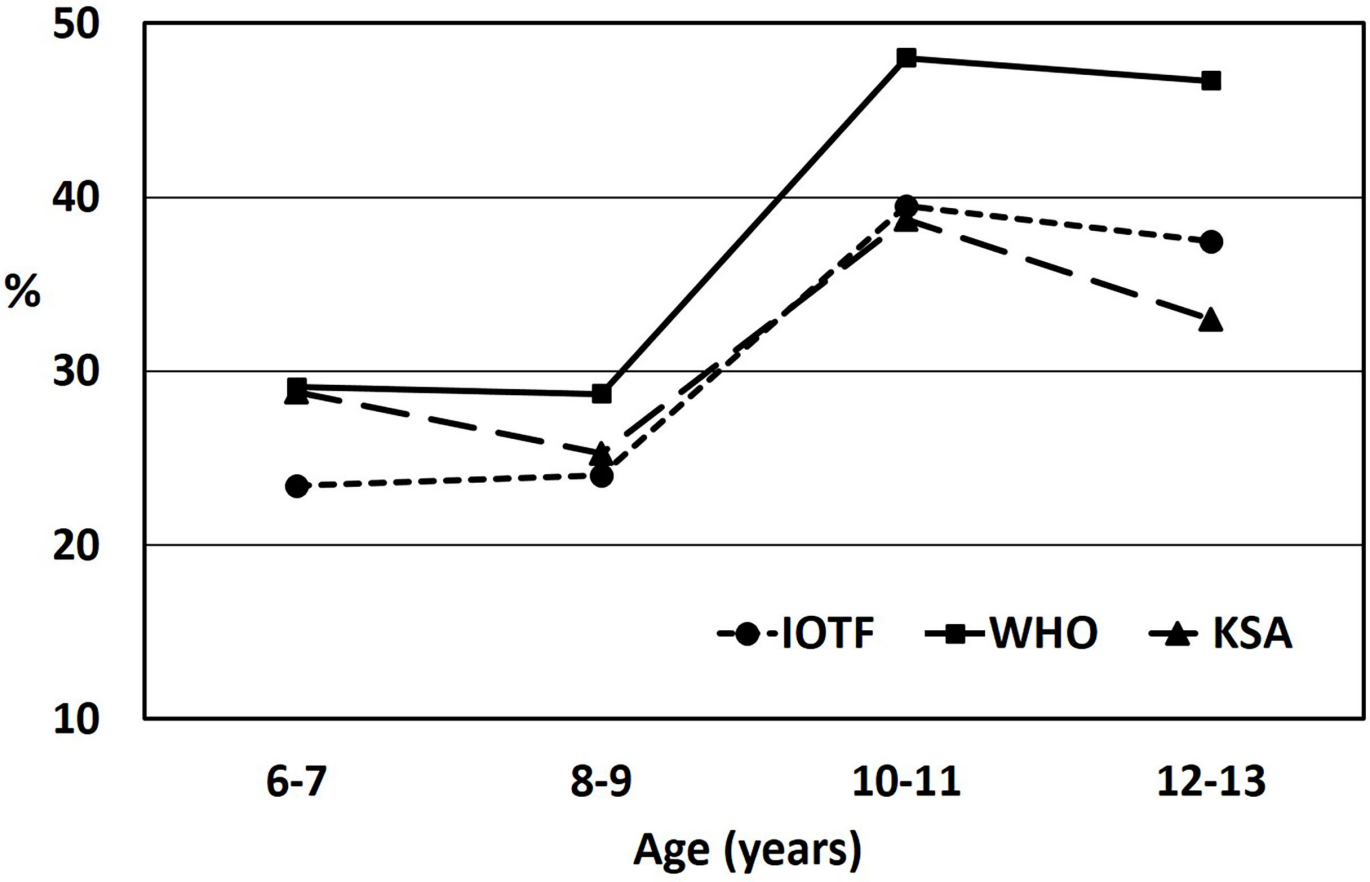
Overweight or obesity prevalence among Saudi children relative to age, based on International Obesity Task Force cut-off standards (IOTF), World Health Organization (WHO) reference standards, or Saudi (KSA) National growth references. Significant differences at *p* < 0.001 in all reference standards were found relative to age.

The proportions (%) of the participants who were classified as underweight, normal weight, overweight, or obese using the IOTF, WHO, and KSA references relative to age are shown in **Table 2**. In general, it was found that the proportion of participants categorized as overweight or obese increased with age when all three classifications were used. Overall, the underweight and obesity prevalence rates were much lower when the KSA reference standards were used than when the other two references were used. Whereas the combined overweight and obesity prevalence was much higher when the WHO reference standards were used. This finding is depicted in **Figure 1**, which shows the prevalence of overweight or obesity by age groups for the three classifications. Also, Spearman’s rho correlation coefficients, while controlling for age, between the three reference standards were fairly high (IOTF reference with WHO reference: r = 0.915, *p* < 0.001; IOTF reference with KSA reference: r = 0.914, *p* < 0.001; and WHO reference with KSA reference: r = 0.866, *p* < 0.001).

**Table 2:** The prevalence of underweight, normal weight, overweight, and obesity among Saudi children using IOTF or WHO reference standards relative to age.

Age groups (years)	Reference standards *	Prevalence (%)			
		underweight	Normal weight	Overweight	Obesity
6	IOTF	26.7	53.5	5.8	14.0
	WHO	26.7	50.0	9.3	14.0
	KSA	5.8	69.8	12.8	11.6
7	IOTF	19.6	55.8	13.2	11.3
	WHO	21.9	47.2	15.8	15.1
	KSA	7.9	61.9	20.4	9.8
8	IOTF	18.8	58.2	12.2	10.8
	WHO	21.5	50.8	11.9	15.7
	KSA	6.6	68.0	18.2	7.2
9	IOTF	14.9	60.1	14.6	10.4
	WHO	19.1	51.2	15.1	14.6
	KSA	7.9	65.9	17.5	8.6
10	IOTF	10.2	48.8	26.5	14.5
	WHO	13.6	39.6	23.5	23.3
	KSA	7.2	50.8	30.3	11.5
11	IOTF	9.4	52.6	23.4	14.6
	WHO	13.0	37.8	27.3	21.9
	KSA	6.5	58.1	25.8	9.6
12	IOTF	7.5	56.1	23.5	12.9
	WHO	12.9	40.0	23.9	23.1
	KSA	5.9	62.4	24.3	7.5
13	IOTF	8.3	47.2	19.5	25.0
	WHO	8.3	47.2	13.9	30.6
	KSA	5.6	52.8	25.0	16.7
**All**	IOTF	13.8	55.1	18.4	12.7
	WHO	17.2	44.8	19.1	18.9
	KSA	7.0	61.3	22.4	9.3

P values of Chi Squares tests for the differences in prevalence categories across ages were < 0.001, < 0.001 and < 0.001 for IOTF, WHO and KSA, respectively.

*Overweight or obesity cut-offs are based on IOTF cut-off values (reference 13), WHO cut-off growth standards (reference 14), or Saudi (KSA) National growth references (reference 22).

**Figure 2:**
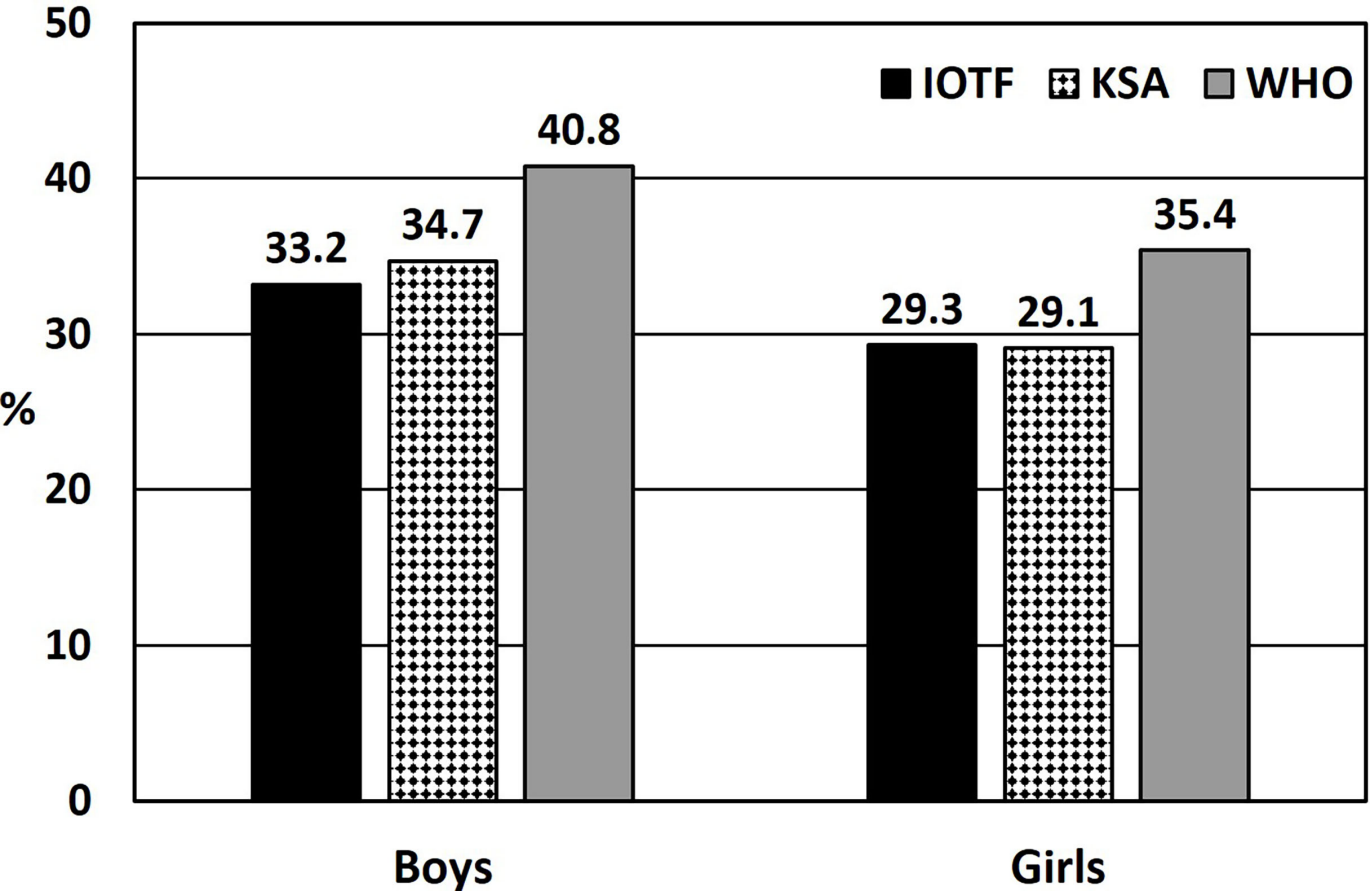
Overweight or obesity prevalence among Saudi children relative to sex, based on International Obesity Task Force cut-off standards (IOTF), World Health Organization (WHO) reference standards, or Saudi (KSA) National growth references. Significant differences between boys’ and girls’ reference standards (*p* values for IOTF = 0.048, WHO = 0.009, and KSA = 0.005).

**Figure 2** illustrates the prevalence of overweight or obesity based on the IOTF, WHO, or KSA references relative to sex. It is clear that, for either sex, the reported overweight or obesity prevalence was higher when the WHO reference standards were used compared to when the IOTF or KSA references were used. There were significant differences found between the boys’ and girls’ reference standards relative to sex (*p* values for the IOTF reference = 0.048, the WHO reference = 0.009, and the KSA reference = 0.005). In addition, Kappa agreement measures for the entire sample between the three reference standards were found to be fairly high; the coefficients were as follows: between the IOTF and KSA references = 0.936 (*p* < 0.001), between the WHO and KSA references = 0.862 (*p* < 0.001), and between the IOTF and WHO references = 0.849 (*p* < 0.001).

**Table 3** displays the prevalence of underweight, normal weight, overweight, and obesity among the participating children when the IOTF, WHO, or KSA reference standards were used relative to selected variables. In each case, the prevalence rates of the BMI categories relative to sex, city, family income, and number of family members living in the house were all significant (*p*-values ranged from < 0.001 to 0.034). However, there was no significant difference between the prevalence rates relative to school type, paternal, or maternal education when any reference standards were used.

**Table 3:** The prevalence of underweight, normal weight, overweight, and obesity among Saudi children using IOTF or WHO reference standards relative to selected variables.

Variable	Reference standards *	Item	Prevalence (%)				*p*-value **
			underweight	Normal weight	Overweight	Obesity	
**Sex**	IOTF	Boys	14.8	52.0	18.0	15.2	0.002
		Girls	12.9	57.8	18.8	10.5
	WHO	Boys	19.3	39.8	17.0	23.8	< 0.001
		Girls	15.3	49.3	20.9	14.4
	KSA	Boys	6.8	58.5	22.4	12.3	< 0.001
		Girls	7.1	63.8	22.5	6.6
**City**	IOTF	Riyadh	12.3	54.0	17.2	16.5	< 0.001
		Jeddah	15.2	56.0	19.5	9.3
	WHO	Riyadh	15.5	44.8	16.8	22.9	< 0.001
		Jeddah	18.8	44.8	21.1	15.3
	KSA	Riyadh	4.9	60.0	22.2	13.0	< 0.001
		Jeddah	8.9	62.5	22.7	6.0
**School type**	IOTF	Public	14.4	55.4	18.2	12.0	0.293
		Private	12.3	54.2	19.1	14.4
	WHO	Public	17.6	45.2	19.0	18.2	0.569
		Private	16.2	43.9	19.3	20.6
	KSA	Public	7.1	62.3	21.3	9.3	0.234
		Private	6.6	58.7	25.3	9.4
**Father’s education**	IOTF	≤ Intermediate	14.9	50.9	18.2	16.0	0.355
		High school	15.4	57.1	16.6	10.9
		University	13.0	55.1	19.2	12.8
		Post graduate	11.6	54.7	20.3	13.4
	WHO	≤ Intermediate	18.6	42.4	17.1	21.9	0.303
		High school	20.1	44.7	18.5	16.7
		University	15.2	45.8	19.9	19.0
		Post graduate	16.2	44.9	18.8	20.1
	KSA	≤ Intermediate	7.4	57.6	21.6	13.4	0.248
		High school	8.0	63.7	20.2	8.1
		University	6.6	60.9	23.4	9.1
		Post graduate	5.6	61.5	24.4	8.5
**Mother’s education**	IOTF	≤ Intermediate	14.4	50.0	19.2	16.3	0.246
		High school	13.8	55.0	18.3	12.9
		University	14.0	56.7	18.2	11.1
		Post graduate	8.6	54.3	18.5	18.5
	WHO	≤ Intermediate	19.9	39.9	15.4	24.8	0.063
		High school	16.8	45.4	19.7	18.2
		University	17.2	46.1	19.5	17.3
		Post graduate	11.1	44.4	21.0	23.5
	KSA	≤ Intermediate	6.7	58.0	22.4	12.8	0.225
		High school	8.0	59.7	23.7	9.1
		University	6.7	63.6	21.5	8.2
		Post graduate	3.7	58.0	25.9	12.3
Family income ***	IOTF	≤ 10,000 SR	15.6	55.2	17.2	12.0	0.032
		10,001-20,000	14.3	57.0	17.3	11.4
		20,001-30,000	11.3	52.4	22.3	14.1
		> 30,001	11.0	47.1	24.3	17.6
	WHO	≤ 10,000 SR	19.7	43.8	19.1	17.4	0.015
		10,001-20,000	17.8	47.0	18.6	16.6
		20,001-30,000	14.0	42.3	20.7	23.0
		> 30,001	13.2	41.9	17.6	27.2
	KSA	≤ 10,000 SR	8.3	61.7	20.6	9.4	0.024
		10,001-20,000	7.5	63.3	21.0	8.3
		20,001-30,000	4.6	58.2	27.8	9.4
		> 30,001	5.8	54.7	25.5	13.9
**Family members living in the house**	IOTF	< 5	15.1	53.3	19.9	11.7	0.022
		5-9	13.6	55.3	17.9	13.1
		10 +	5.9	68.3	10.9	14.9
	WHO	< 5	18.1	43.3	21.1	17.5	0.034
		5-9	17.3	45.1	17.9	19.7
		10 +	9.9	58.4	13.9	17.8
	KSA	< 5	7.3	60.9	23.8	8.1	0.077
		5-9	7.3	61.0	21.9	9.9
		10 +	2.0	72.3	14.9	10.9

*Overweight or obesity cut-offs are based on IOTF cut-off values (reference 13), WHO growth cut-off standards (reference 14), or Saudi growth references – KSA (reference 22).

**Differences in proportions between the selected variable items and prevalence rate.

**In Saudi Riyal (US $ = 3.75 Saudi Riyal).

**Table 4** shows the logistic regression analysis results, adjusted for age, for selected sociodemographic variables relative to overweight/obesity versus non-overweight/non-obesity among the participants. There were significant associations (*p* < 0.001) between overweight/obesity and increasing age when all three classification standards were used. However, only when the WHO (*p* = 0.043) and KSA (*p* = 0.036) standards were used was there a significant association between overweight/obesity and sex (boys). In terms of geographic region, the incidence of overweight/obesity compared to the incidence of non-overweight/non-obesity was higher in children living in Riyadh than in children living in Jeddah when the references from the IOTF (*p* = 0.031) and KSA (*p* = 0.005) were used, but not when the WHO reference was used (*p* = 0.122). Furthermore, having a small family (2–5 members) in the house was associated with increased prevalence of overweight/obesity irrespective of the classification system (*p* values ranged from 0.017 to 0.025). Parental education levels did not show any significant association with overweight/obesity in all classification systems. Finally, a low to intermediate family income of (10,001–20,000 Saudi Riyals) was found to be significantly (*p* = 0.015) associated with overweight/obesity when using the IOTF standards.

**Table 4:** Results of logistic regression analysis, adjusted for age, of selected sociodemographic variables relative to overweight/obesity versus non-overweight/non-obesity among Saudi children.

Variable		Overweight/obesity versus non-overweight/non-obesity *						
		IOTF			WHO		KSA	
		aOR (95% CI)		***p-*** *value*	aOR (95% CI)	***p-*** *value*	aOR (95% CI)	***p-*** *value*
**Age**		1.203 (1.138-1.272)	< 0.001	1.246 (1.181-1.315)	< 0.001	1.107 (1.048-1.169)	< 0.001	
**Sex** (girls = ref)		1.00		1.00		1.00		
Boys		1.136 (0.938-1.374)	0.191	1.207 (1.006-1.448)	0.043	1.223 (1.013-1.477)	0.036	
**City** (Jeddah = ref)		1.00		1.00		1.00		
Riyadh		1.258 (1.021-1.552)	0.031	1.171 (0.958-1.432)	0.122	1.344 (1.092-1.654)	0.005	
**School type** (private = ref)		1.00		1.00		1.00		
Public		0.900 (0.722-1.122)	0.349	0.902 (0.729-1.114)	0.338	0.871 (0.700-1.082)	0.212	
**Members of Family in the house** (‗ 10 = ref)		1.00		1.00		1.00		
6-9		1.537 (0.946-2.500)	0.083	1.468 (0.930-2.319)	0.099	1.556 (0.960-2.522)	0.072	
2-5		1.823 (1.105-3.007)	0.019	1.779 (1.111-2.849)	0.017	1.764 (1.073-2.899)	0.025	
**Father education** (postgraduate = ref)		1.00		1.00		1.00		
College degree		1.024 (0.740-1.417)	0.885	1.058 (0.774-1.447)	0.723	1.097 (0.794-1.515)	0.574	
High school		0.780 (0.544-1.118)	0.176	0.869 (0.616-1.227)	0.426	0.868 (0.607-1.241)	0.437	
< Intermediate		1.020 (0.656-1.585)	0.930	0.986 (0.644-1.510)	0.950	1.127 (0.728-1.744)	0.593	
**Mother education** postgraduate = ref)		1.00		1.00		1.00		
College degree		0.736 (0.450-1.204)	0.223	0.765 (0.473-1.238)	0.275	0.700 (0.431-1.138)	0.150	
High school		0.826 (0.494-1.381)	0.465	0.796 (0.481-1.316)	0.373	0.800 (0.482-1.329)	0.389	
< Intermediate		1.019 (0.582-1.785)	0.947	0.912 (0.528-1.577)	0.742	0.920 (0.529-1.599)	0.766	
**Family income** > 30,001 = ref)		1.00		1.00		1.00		
20,001-30,000		0.874 (0.560-1.283)	0.434	1.001 (0.664-1.508)	0.997	0.959 (0.634-1.451)	0.842	
10,001-20,000		0.614 (0.414-0.909)	0.015	0.724 (0.491-1.067)	0.103	0.696 (0.470-1.032)	0.071	
< 10,000		1.696 (0.455-1.065)	0.095	0.839 (0.553-1.273)	0.409	0.820 (0.536-1.253)	0.359	

*Non-overweight/non-obesity was used as a reference category. aOR, age adjusted odds ratio; CI, confidence interval; ref, reference category.

IOTF: International Obesity Task Force age- and sex-specific BMI cutoff reference standards. WHO: World Health Organization growth references for school-aged children and adolescents. KSA: Saudi National growth references calculated from the z−scores of BMI for age for children and adolescents from 5 to 18 years.

## Discussion

The present study aimed to compare the results of three different BMI classifications (the IOTF age and gender cutoffs, the WHO growth references for school-aged children, and the KSA national growth references) using data obtained from Saudi children aged 6–13 years. The findings showed that the proportions of the participants classified as overweight or obese were fairly high, regardless of the classification system used. The IOTF cutoffs appear to be somewhat closer to the KSA growth references than to the WHO references. In all three classifications, girls exhibited lower overweight or obesity prevalence than boys. Family income, but not paternal or maternal education, was significantly associated with overweight/obesity when the IOTF standards were used. In addition, having a small family in the house was significantly associated with obesity, irrespective of the reference used. Hence, it seems that estimating the prevalence of underweight, overweight, and obesity among Saudi children yields inconsistent results when the IOTF, WHO, and KSA growth references are used.

Regardless of the BMI reference standards used, we observed a high prevalence of overweight and obesity among the participants, with somewhat variable levels of underweight status. It appears that the prevalence of overweight and obesity among Saudi children and adolescents has been rising over the last decades (9, 10). Recently, it was observed that the most important risk factors for obesity among Saudi children 5–9 years-of-age are parental characteristics, awareness of the degree of obesity burden, and lifestyle behaviors, such as frequent snacking, physical inactivity, and screen time (27). Also, among Saudi children and adolescents, obesity and other risk factors were found to have a significant impact on abnormal glucose metabolism (28). Therefore, efforts to prevent overweight and obesity in children must focus primarily on early identification, followed by appropriate reduction of common risk factors.

The present study found a higher prevalence of underweight, overweight, and obesity when the WHO reference standards were used compared to the IOTF cutoffs and the KSA national growth references. Currently, the use of age- and gender-specific BMI cutoffs is recommended to estimate overweight and obesity status among children and adolescents (13, 14, 29). However, it is somewhat challenging to estimate overweight and obesity prevalence when the most common international classification systems reveal different results (15–20, 30, 31). It appears that the discrepancies resulting from the use of the IOTF and WHO reference standards are due to differences in the cutoff values, the criteria used to select the sample, and the approaches used to define the cutoffs (32). An understanding of how the IOTF and WHO BMI standards for children and adolescents are constructed and their comparability may provide an explanation of their inherent limitations. In contrast to adult anthropometric cutoffs, which are based on mortality outcomes (33, 34), BMI cutoffs for children under the age of 18 years are statistically determined (13, 14). Indeed, it was reported that using the IOTF cutoffs and population-specific standards for childhood BMI failed to adequately predict cardiovascular disease risk factors in mid-adulthood from childhood BMI values (35). Accordingly, the choice of the reference standards used to express BMI data may influence the status of overweight and obesity among children from different populations. In addition, such differences in overweight and obesity, based on various cut-off references, may impact policy decision-making. In light of such limitations, many studies have argued that common references cannot be applied to children from different populations since they differ in their growth patterns (36–38). However, from the findings of the present study, it appears that the agreement between the IOTF and KSA references is much closer than that between the WHO and KSA references, when defining overweight plus obesity prevalence among Saudi children.

In terms of agreement measures, the Kappa agreement measures among the three reference standards were found to be fairly high, with the coefficients ranging from 0.936 between the IOTF and KSA references to 0.849 between the IOTF and WHO references. A lower Kappa coefficient (0.72) was reported between the IOTF and WHO references in a study with Brazilian children (39). Moreover, agreement between the IOTF and WHO references and French references ranged from moderate (Kappa = 0.43) to perfect (Kappa = 1.00) among French children (40). However, in a group of South American children, moderate agreements were observed between body fat estimated by dual-energy x-ray absorptiometry (DXA) and by the IOTF (Kappa = 0.61) and WHO (Kappa = 0.63) references, with the IOTF cutoffs showing the highest specificity (0.98 [0.94, 0.99]) (41). Data from a study conducted with Italian children and adolescents aged 5–17 years indicated that the WHO references had the highest sensitivity, while the IOTF classification had the highest specificity, in identifying obese subjects with clustered cardiometabolic risk factors (42).

Previous local, regional, and international studies have reported varying prevalence rates of overweight and obesity among children and adolescents when using the IOTF or WHO reference standards. Among Saudi adolescents from Riyadh, the IOTF reference reportedly produced more conservative (by 4–6%) estimates of overweight and obesity than the WHO reference standards (43). The average difference in overweight/obesity prevalence when using IOTF and WHO references in our study was 6.9%. In another study conducted on 6–16-year-old school children from Riyadh, the overall prevalence rates of overweight and obesity, as defined by the WHO 2007 growth standards, were reported to be 13.4% (14.2% for girls and 12% for boys) and 18.2% (18% for girls and 18.4% for boys), respectively (11). In comparison, in the present study with children aged 6–13 years, the overweight prevalence was higher (19.1%), but the obesity rate was similar (18.9%).

At the regional level, among a group of 10–14-year-old Kuwaiti adolescents, the prevalence of overweight and obesity calculated using Kuwaiti local reference data (36.7%) was significantly lower than that obtained using the IOTF (44.7%) or WHO (50.5%) reference standards (44). In another study, the prevalence of overweight and obesity among school children aged 10–13 years in Bahrain was calculated to be higher when the WHO reference was used compared to when the IOTF reference was used (17). Also, a school-based cross-sectional study conducted in eight Arab countries, including Saudi Arabia, involving adolescents aged 15–18 years showed that the use of the WHO standard resulted in a lower prevalence of overweight but a higher prevalence of obesity than the use of the IOTF reference standards (45).

Internationally, studies have shown varying degrees of consistency. Among 5–17-year-old Canadian children and adolescents, 16.4% of the participants were classified as overweight and 8.4% as obese when the IOTF reference was used, while 19.8% were classified as overweight and 11.7% as obese when the WHO standards were used (18). Moreover, the IOTF classification appears to be more specific when applied to identify overweight and obesity among indigenous Canadian school children aged 8–14 years than other systems, such as those of the Centers for Disease Control and Prevention (CDC) and the WHO (15). A recent study involving Cree youth revealed that participants classified as overweight by the IOTF classification system, but not by the WHO reference standards, displayed less severe clinical obesity (20). That is, false-positive subjects with obesity identified by WHO cutoffs were effectively classified as overweight by IOTF (20). Inconsistency was also apparent when the prevalence of underweight, overweight, and obesity among Malaysian children aged 6–14 years was estimated; use of the WHO reference resulted in a higher prevalence of overweight and obesity than the use of the IOTF reference (31). Thus, from previous studies’ findings, it appears that the IOTF reference standards are more accurate than those of the WHO in identifying children and adolescents with obesity (15–20, 38).

Paternal or maternal education levels in the current study did not show any significant association with overweight/obesity in all classification systems. However, family income exhibited a significant association with overweight/obesity when the IOTF standards were used. However, when the WHO 2007 growth standards were used, overweight and obesity among school children from Riyadh aged 6–16 years appeared to significantly increase with higher socioeconomic status, including higher family income (11). In a study involving adolescents from eight Arab countries, including Saudi Arabia, major differences in obesity prevalence were found among the eight countries when both the IOTF and WHO reference standards were used, and the differences were attributed to a variety of factors, including socioeconomic status (45). Also, in a population-based cross-sectional study involving Pakistani children aged 5–12 years, a significant correlation was found between overweight and obesity status and high socioeconomic status, whereas body thinness was associated with low socioeconomic status and lower parental education (30). Interestingly, an analysis of data from a large number of children aged 6–9 years in 24 countries in the WHO European region showed that there was an inverse relationship between the prevalence of childhood overweight or obesity and parental education in high-income countries, and a positive correlation was observed in most of the middle-income countries (46).

Across all three classification systems used in this study, girls exhibited lower overweight or obesity incidence than boys. This finding aligns with results reported in some previous studies that used the IOTF or WHO cutoff references (10, 31, 47). However, others have reported mixed results (11, 15, 17, 20, 38). Finally, our findings revealed that having a small family in the house was significantly associated with increased prevalence of overweight/obesity irrespective of the reference used. This is an important finding of the present study. A recent study from the United States indicated that having more siblings is associated with lower BMI and decreased likelihood of obesity (48). It may be speculated that larger families may have a bigger reason to prepare and eat meals at home, which means better meal quality for children. Also, small families may be more inclined (and can afford) to eat outside home, which may include more fast foods. Another confounding factor for the relationship between obesity and family size may include family income, however, the correlation between family size and income was weak in the present study. It is possible that physical activity and dietary intake may influence body weight, but we did not assess these two factors in the present study.

The present study has some strengths and limitations. The strengths of this study include a relatively large sample size and representative BMI data from children in two major Saudi cities. Also, measurements of weight and height were performed directly and did not rely on self-reporting. In addition, the sample was drawn from both public and private schools. The present study, however, has some limitations. First, the findings are limited to children aged 6–13 years and cannot be generalized to adolescents aged 14–17 years or preschoolers from 2-5 years. Second, the sample was drawn from urban areas and cannot be generalized to children residing in rural areas.

## Conclusion

The proportions of the Saudi children who were classified as overweight or obese appeared to be fairly high, regardless of the classification system used. The IOTF cutoffs appear to be somewhat closer to the KSA growth references than to the WHO references. The Kappa agreement measures between the three references were found to be high, with the coefficients ranging from as high as 0.936 (between the IOTF and KSA references) to as low as 0.849 (between the IOTF and WHO references). In all three classifications, girls exhibited a lower incidence of overweight or obesity compared with boys. Family income, but not parental or maternal education, exhibited a significant positive association with obesity when using the IOTF standards. In addition, having a large family in the house was significantly associated with decreased obesity, irrespective of the IOTF or WHO references. It seems that using the IOTF, WHO, or KSA growth references to estimate the prevalence of underweight, overweight, and obesity among Saudi children leads to inconsistent results. However, the agreement between the IOTF and KSA references is much closer than that between the WHO and KSA references, which means that the IOTF classification system performed in a similar way to the KSA references (31.1% versus 31.7%) compared to the WHO references (38.0%) when assessing the overall prevalence of overweight plus obesity among Saudi children. Therefore, the choice of the currently available BMI classification systems has important implications for child health and the assessment of clinical obesity.

## Data availability statement

The original contributions presented in the study are included in the article/supplementary material. Further inquiries can be directed to the corresponding author.

## Ethics statement

Ethical approval was obtained from the Institutional Review Board (IRB) at King Saud University, Riyadh (IRB Log Number: 17/0064/IRB) and Princess Nourah bint Abdulrahman University, Riyadh (IRB Log Number: 19-0014). Written informed consent to participate in this study was provided by the participants‘ legal guardian/next of kin.

## Author contributions

Conceptualization: HA-H. Methodology: HA-H, AMA, MA, AAA, LJ, and RA. Investigation: HA-H, AMA, MA, AAA, LJ, RA, RB, and SA. Data collection and supervision: HA-H, AMA, MA, AAA, LJ, and RA. Statistical analysis: HA-H. Interpretation of the findings: HA-H, AMA, MA, AAA, LJ, RA, RB, and SA. Drafting the paper: HA-H. Reviewing and editing the draft: AMA, MA, AAA, LJ, RA, RB, and SA. All authors critically read, revised the draft for important intellectual content, approved the final version of the manuscript to be published, and agreed to be accountable for all aspects of the work.

## Conflict of interest

The authors declare that the research was conducted in the absence of any commercial or financial relationships that could be construed as a potential conflict of interest.

## Publisher’s note

All claims expressed in this article are solely those of the authors and do not necessarily represent those of their affiliated organizations, or those of the publisher, the editors and the reviewers. Any product that may be evaluated in this article, or claim that may be made by its manufacturer, is not guaranteed or endorsed by the publisher.
