# Corrigendum: Prevalence of overweight and obesity among saudi children: a comparison of two widely used international standards and the national growth references

**DOI:** 10.3389/fendo.2023.1305460

**Published:** 2023-11-02

**Authors:** Hazzaa M. Al-Hazzaa, Amani A. Alrasheedi, Rayan A. Alsulaimani, Laura Jabri, Abdulrahman M. Alhowikan, Maha H. Alhussain, Rowaedh A. Bawaked, Saleh A. Alqahtani

**Affiliations:** ^1^ Lifestyle and Health Research Center, Health Sciences Research Center, Princess Nourah Bint Abdulrahman University, Riyadh, Saudi Arabia; ^2^ Department of Food and Nutrition, Faculty of Human Sciences and Design, King Abdulaziz University, Jeddah, Saudi Arabia; ^3^ Department of Pharmacology, Faculty of Medicine, King Abdulaziz University, Jeddah, Saudi Arabia; ^4^ American International School of Jeddah, Jeddah, Saudi Arabia; ^5^ Department of Physiology, College of Medicine, King Saud University, Riyadh, Saudi Arabia; ^6^ Department of Food Science & Nutrition, College of Foods & Agricultural Sciences, King Saud University, Riyadh, Saudi Arabia; ^7^ Department of Public Health, College of Health Sciences, Saudi Electronic University, Riyadh, Saudi Arabia; ^8^ Division of Gastroenterology and Hepatology, Johns Hopkins University, Baltimore, MD, United States; ^9^ King Faisal Specialist Hospital & Research Center, Riyadh, Saudi Arabia

**Keywords:** body mass index (BMI), children, International Obesity Task Force (IOTF), overweight, obesity, sociodemographic, underweight, World Health Organization (WHO)

**Incorrect Affiliation**

In the published article, there was an error in affiliation 2 and 3. For Affiliation 2, instead of “Food and Nutrition Department, College of Human Sciences and Design, King Abdul Aziz University, Jeddah, Saudi Arabia” it should be “Department of Food and Nutrition, Faculty of Human Sciences and Design, King Abdulaziz University, Jeddah, Saudi Arabia”. For affiliation 3, instead of 3Department of Pharmacology, Faculty of Medicine, King Abdul Aziz University, Jeddah, Saudi Arabia” it should be “Department of Pharmacology, Faculty of Medicine, King Abdulaziz University, Jeddah, Saudi Arabia”.

The authors apologize for this error and state that this does not change the scientific conclusions of the article in any way. The original article has been updated.

